# Description of normal pulmonary radiographic findings in 55 apparently healthy juvenile Kemp's ridley sea turtles (*Lepidochelys kempii*)

**DOI:** 10.3389/fvets.2023.1101206

**Published:** 2023-02-06

**Authors:** Christa E. Barrett, Debra P. Moore, Alison M. Lee, Sophie Dennison

**Affiliations:** ^1^Global Center for Aquatic Health & Food Security, Mississippi State University, Mississippi State, MS, United States; ^2^Department of Clinical Sciences, College of Veterinary Medicine, Mississippi State University, Mississippi State, MS, United States; ^3^TeleVet Imaging Solutions, PLLC, Oakton, VA, United States

**Keywords:** Kemp's ridley, *Lepidochelys kempii*, sea turtle, radiography, pulmonary, lungs

## Abstract

A total of 55 digital radiographic studies from 53 individual juvenile Kemp's ridley sea turtles (*Lepidochelys kempii*) were retrospectively used to determine the normal radiographic anatomy of the lower respiratory tract in sea turtles that had been stranded due to hook-and-line injury and were otherwise clinically healthy. There were three or four projections available for each study: dorsoventral (DV), rostrocaudal (RoCd), and left and/or right lateral. The DV and RoCd were most conducive for assessing global lung volume and symmetry of lung volume. The DV and lateral views were most helpful for evaluating the main bronchus and its branching channels and for assessing lung margination. The RoCd view was most useful for assessing the symmetry of the lung opacity. The lateral views were most helpful for assessing the ventral margin of each lung lobe. On the lateral view, the main bronchus lay ventrally and coursed horizontally through the lung from cranial to caudal. On the DV view, the bronchus lay medially and was observed to be curvilinear coursing caudomedially. On the RoCd view, the main bronchus was located ventromedially. The RoCd view demonstrated the channels and niches end-on resulting in a reticulated or honeycomb appearance. The channels were seen as uniform striations coursing perpendicular to the main bronchus on the lateral views (vertical striations coursing dorsal to ventral) and DV views (horizontal striations coursing medially to laterally).

## Introduction

Kemp's ridley sea turtles (*Lepidochelys kempii*) are the smallest sea turtle worldwide and a critically endangered species found in oceans. They usually inhabit the southern United States, Mexico, and, seasonally, oceans as far north as New England regions of the United States. Evidence in 2014 documented that only ~4,800 nesting females were identified in popular nesting sites in Mexico (1).

When *Chelonia* species become ill, they often show non-specific clinical signs for a variety of diseases. In most cases of injured or stranded sea turtles, physical examination alone does not provide sufficient information, and radiography is the most used diagnostic modality for the evaluation of the respiratory tract of sea turtles.

Respiratory disease is a prominent and important diagnostic finding in sea turtles (2). Chelonians lack a functional diaphragm and cannot cough, making clearance of infections and inflammatory debris within the lungs nearly impossible (3). In a retrospective study of cold-stunned Kemp's ridley sea turtles in rehabilitation facilities, diseases of the respiratory system including bacterial and fungal diseases were one of the most common findings (2).

Several authors concluded that the inhalation of water is possibly one of the primary causes of lung infections (4). A recent National Oceanic and Atmospheric Administration (NOAA) report, investigating sea turtle stranding and mortality efforts from 2010 to 2019 in the Northern Gulf of Mexico, noted that 49% of all mortality was likely related to drowning. Forced submergence in fisheries was suspected based on necropsy findings and exclusion of other causes (5). Causes of respiratory disease due to forced submergence include drowning and the development of gas embolism often caused by trawling (6). In addition, foreign bodies such as fishing hooks may easily involve the trachea and bronchi through ingestion into the oral cavity and esophagus (4). Incidental hook-and-line capture is the most common presentation of Kemp's ridley sea turtles to the stranding network in Mississippi. From 2010 to 2014, over 800 sea turtles were incidentally captured by fishing hooks used by recreational anglers in the Mississippi Sound, USA, off coastal piers (7). Trauma due to boat strikes is another common cause of respiratory disease, and the dorsal location of the lungs within the coelomic cavity makes them particularly susceptible to injury (8).

Radiographic abnormalities in the lungs of cold-stunned Kemp's ridley sea turtles, normal cervical and coelomic radiologic features of loggerhead sea turtles (*Caretta caretta*), and an in-depth computed tomographic (CT) study for the normal structure of the lungs of the loggerhead sea turtle have been described; however, there is no descriptive analysis outlining the normal radiographic anatomy of pulmonary structures using digital radiography in the Kemp's ridley sea turtle species (9–11). Sea turtles selected for this review were incidentally caught by hook-and-line off of fishing piers in the Mississippi Sound, MS while being clinically and diagnostically unharmed. The purpose of this article is to provide descriptions of normal radiographic anatomy of the lower respiratory tract of apparently healthy juvenile Kemp's ridley sea turtles.

## Methods

Sea turtle rehabilitation at the Institute for Marine Mammal Studies (IMMS) is partially supported by funding secured by the Mississippi State University (MSU) Global Center for Aquatic Health and Food Security (GCAHFS) for the Mississippi Marine Mammal and Turtle Conservation, Recovery, and Monitoring Program, which is funded by the National Fish and Wildlife Foundation under Mississippi Department of Environmental Quality Agreement No. 18-00081 and is conducted under the authorization of the United States Fish and Wildlife Service and the Mississippi Department of Marine Resources. Permits for rehabilitation services were obtained from the US Fish and Wildlife Services permit numbers TE12392A-2 (effective 8/11/2016-7/31/2019) and TE12392A-3 (10/29/2020-10/31/2025).

On intake into IMMS' rehabilitation facility, sea turtles are weighed and examined by an MSU veterinarian in and out of the water, and radiographs, blood, and morphometric measurements are obtained by IMMS' stranding technicians and/or veterinary technicians. Radiographs are evaluated by an MSU or another board-certified veterinary radiologist. Routine blood work includes an in-house packed cell volume (PCV) and total solids (TS), as well as sending blood samples to an outside laboratory capable of performing a reptile complete blood count (CBC) and reptile plasma biochemical analysis, which is then evaluated by an MSU veterinarian. Age and sex cannot be determined externally for immature sea turtles; because of this, they are put into age groups based on the straight carapace length (SCL). Straight carapace lengths are measured with a large caliper as a straight line from the nuchal notch of the animal to the posterior end/notch of the posterior marginal scutes. Kemp's ridley sea turtles with a straight carapace length of <40 cm are classified as juveniles.

In this retrospective study, radiographs from all juvenile Kemp's ridley sea turtles that were brought to this rehabilitation facility due to incidental hook-and-line capture from the state of Mississippi between 2016 and 2020 were all evaluated independently by two board-certified veterinary radiologists (DACVRs). Sea turtles of other species were not included and neither were Kemp's ridley sea turtles with straight carapace lengths measuring over 40 cm. Any sea turtles with pulmonary abnormalities or a history of respiratory diseases (such as nasal discharge) were excluded. Medical records of all the remaining cases were reviewed by an aquatic veterinarian, and cases were excluded if there was evidence of significant trauma (e.g., vessel strike, and predation), buoyancy issues in the water, hematologic and plasma biochemical abnormalities, or other comorbidities. Cases that remained were deemed healthy juvenile Kemp's ridley sea turtles other than the hook-and-line capture.

For the majority of turtles, dorsoventral (DV), rostrocaudal (RoCd), and right and left laterals were evaluated. Exceptions for these projections included turtles between the years 2016 and 2017, which were limited to one lateral projection for each animal, and one turtle from 2018 in which a RoCd projection was not included. All projections attempted to achieve a distance as close to 40 cm as possible between the radiographic cassette and the radiogenic source. DV projections were obtained using a vertical beam orientation at 68 kV and 7.0 mAs. RoCd and lateral projections were obtained using a horizontal beam orientation at 78 kV and 7.0 mAs. Turtles were positioned for each projection as described by Pease et al. (12). All radiographic images were acquired *via* digital direct radiography (DR) using Quantum Medical Imaging, Quest HF Series (Quantum Medical Imaging, LLC, Ronkonkoma, NY 11779 USA) radiograph machine with a Trixell, and Pixium FE 3543 pR (Trixell, Moirans, France) cassette, and images were archived to Asteris Keystone picture archiving and communication system (PACS).

All radiographs were assessed independently and blindly by two American College of Veterinary Radiology-board certified veterinary radiologists (SD and AL), who both have extensive sea turtle imaging experience, to ensure images were of diagnostic quality and included all anatomy. The two DACVRS have previously worked collaboratively on IMMS turtle cases with a strong agreement; thus, the total number of cases was divided equally between the two DACVRs for evaluation. Any questionable studies were re-evaluated and a consensus was reached. The DACVRs had the freedom to choose when and how many studies were read in each session within an assigned 2-week period, and studies could be compared to reference studies within each DACVRs' personal library or from the literature as needed. Operator-selected windowing and leveling were used to optimize the images in all cases with the freedom to zoom in and pan the images. The pulmonary parenchyma appearance, symmetry, opacity, and volume were assessed with an expectation that the main bronchus and adjacent arteries and veins should be easily identifiable and surrounded by lucent, uniform-appearing lungs. Any regions of increased parenchymal opacity or decreased conspicuity of the main bronchus wall or the margins of the main lobar artery and vein were noted, with particular focus on location (left/right and dorsal/ventral) and distribution (diffuse, focal, and multifocal). Lung volume was assessed, with an expectation that the lung would occupy ~40 ± 10% of the cross-sectional area of the shell on the RoCd projection. Any asymmetry or subjective decreased volume was noted. Where evident, a cause for decreased volume was stated (for example, gastrointestinal tract distension). Finally, the lung margin was assessed for any deep indentions or asymmetrical irregularities and any evidence of thickening of the pleura.

## Results

Fifty-five radiographic studies from 53 individual healthy juvenile Kemp's ridley sea turtles met the criteria for inclusion. Two of the 55 radiographic studies were recaptures, resulting in 53 individual cases. All 53 sea turtles survived to release. A total of 26 cases were interpreted by AL and 27 cases by SD. No questionable observations that required consensus review were identified.

**Figure 1:**
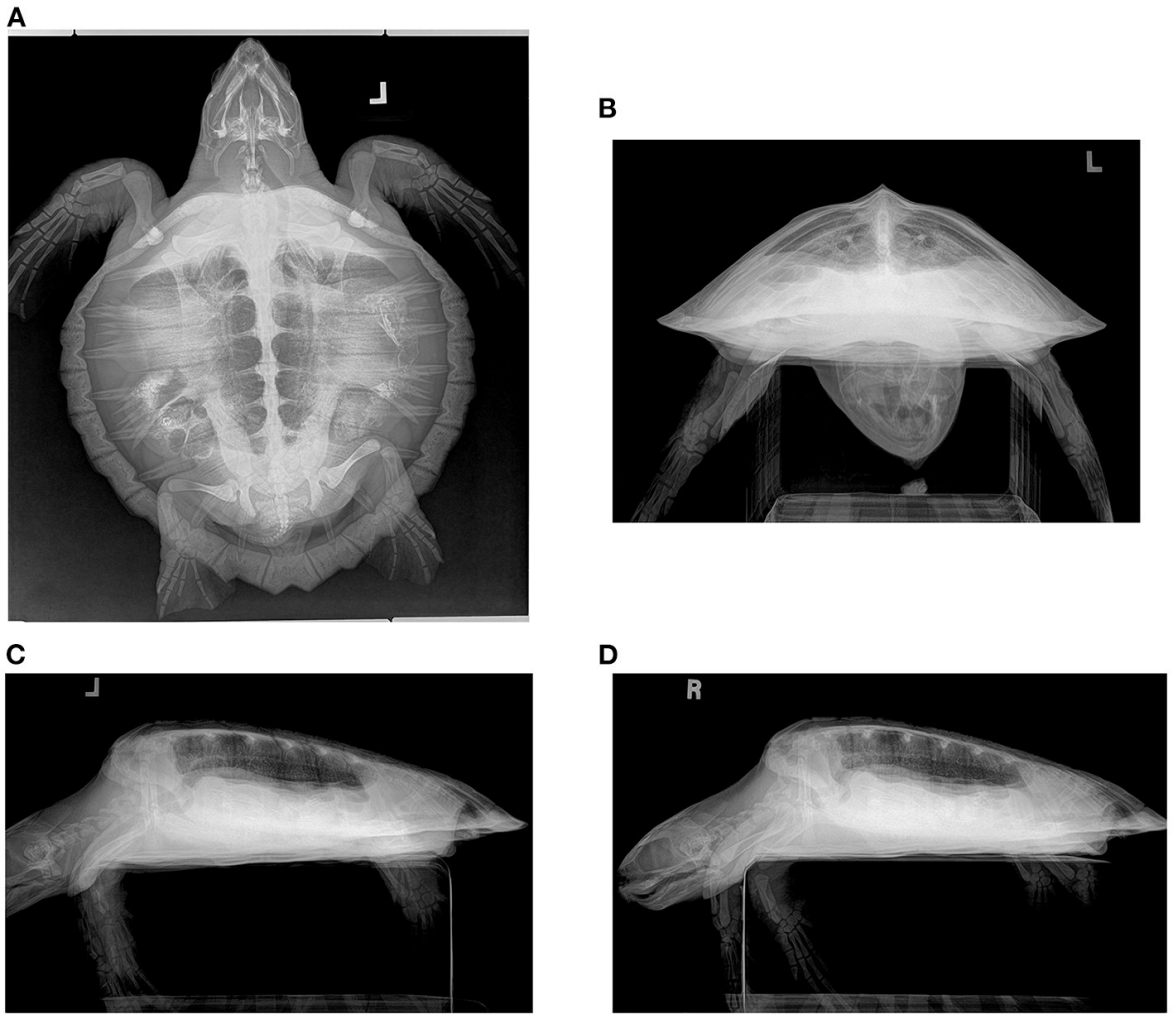
Radiograph series in a hook-and-line capture but otherwise healthy juvenile Kemp's ridley sea turtle (*Lepidochelys kempii*). **(A)** Dorsoventral (DV) projection: The trachea is seen left of the midline in the cervical region before bifurcating in the cranial central coelom. The lung field is seen; however, superimposition of gastrointestinal ingesta and gas present within the gastrointestinal tract limits the full assessment of some regions. The pulmonary artery (lateral) and pulmonary vein (medial) can be seen in parallel coursing through the lung on either side of the bronchus. **(B)** Rostrocaudal (RoCd) projection: Both lung fields are relatively symmetric making up ~30% of the coelomic cavity. The main stem bronchus is evident in each central lung represented by a thin-walled radiopaque circular structure that is radiolucent in the center. The pulmonary arteries can be seen just dorsal to the main bronchus. Less evident are the pulmonary veins ventral to the main bronchus. **(C)** Left and **(D)** Right Lateral Projection: The lung margins are smooth, and the lung fields occupy ~40 ± 10% of the coelomic cavity. The main bronchus is end-on. The pulmonary arteries are dorsal to the main bronchus and the pulmonary veins are ventral, both seen slightly obliquely in this view. On the right lateral projection, gas-distended gastrointestinal loops are seen partially superimposed on the ventral lung fields.

**Figure 2:**
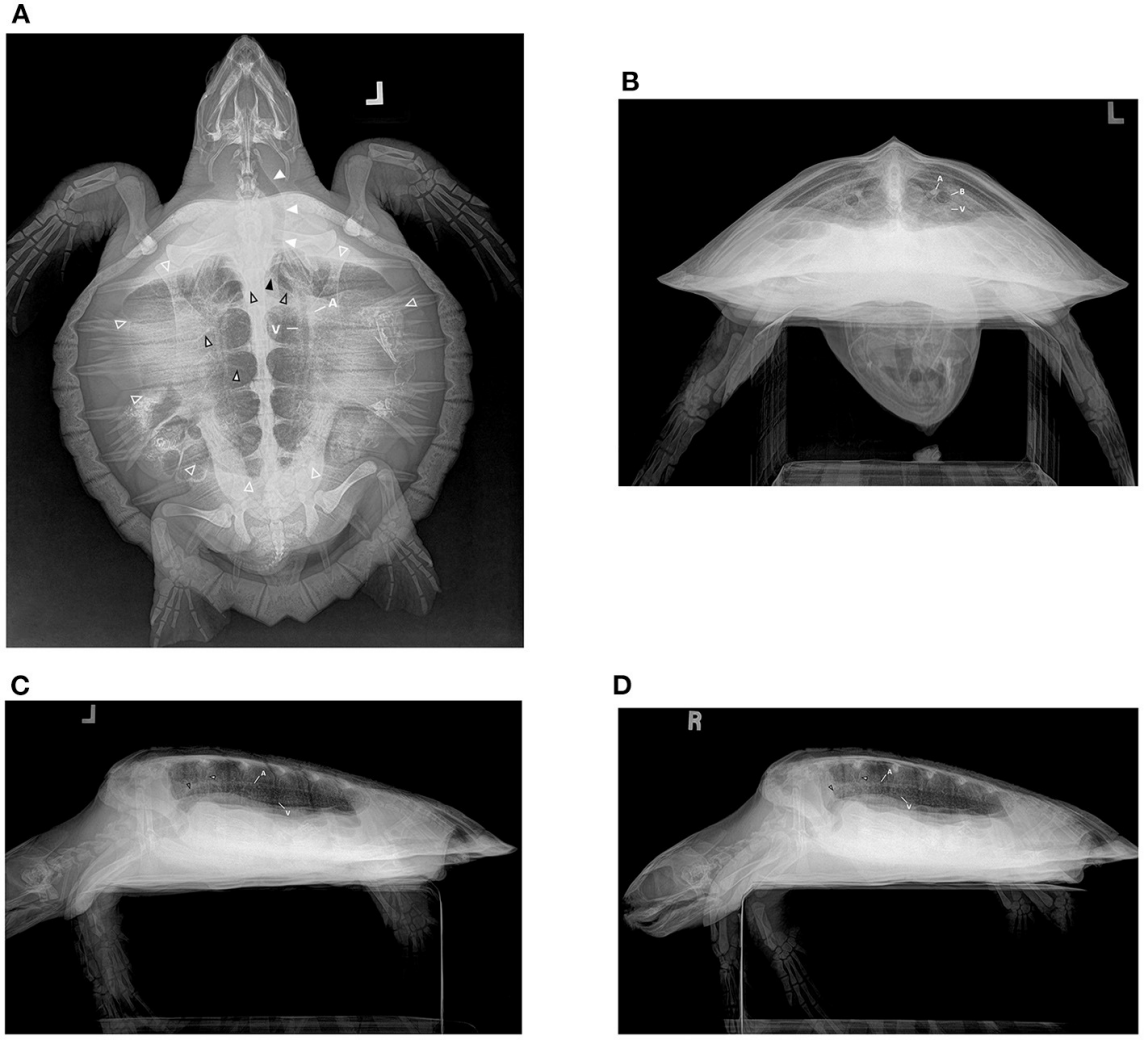
Radiograph series in a hook-and-line capture but otherwise healthy juvenile Kemp's ridley sea turtle (*Lepidochelys kempii*). **(A)** Dorsoventral (DV) projection: The trachea is seen left of midline (solid white arrows) in the cervical region before bifurcating (solid black arrow) in the cranial central coelom into the right and left main bronchus (black outlined hollow arrows). The main bronchus is seen coursing from cranial to caudal in the medial lung field as horizontal soft tissue opaque striations of branching channels (black outlined white arrows) are observed coursing from the mainstem bronchus to the periphery perpendicular to the main bronchus. The lung field is seen (while outlined hollow arrows); however, superimposition of gastrointestinal ingesta and gas present within the gastrointestinal tract limits the full assessment of some regions. The pulmonary artery (A) and pulmonary vein (V) can be seen in parallel coursing through the lung on either side of the bronchus. **(B)** Rostrocaudal (RoCd) projection: Both lung fields are relatively symmetric making up ~30% of the coelomic cavity. The mainstem bronchus (B) is evident in each central lung represented by a thin-walled radiopaque circular structure that is radiolucent in the center. The pulmonary arteries (A) can be seen just dorsal to the main bronchus. Less evident are the pulmonary veins (V) ventral to the main bronchus. **(C)** Left and **(D)** Right Lateral Projection: The lung margins are smooth, and the lung fields occupy ~40 ± 10% of the coelomic cavity. The main bronchus (black outlined hollow arrow) is end-on and is seen coursing from cranial to caudal, horizontally through the ventral lung field. There are vertical striations (black outlined white arrow) through the lungs on these views that reflect the channels that branch from the main bronchus. The pulmonary arteries (A) are dorsal to the main bronchus and the pulmonary veins (V) are ventral, both seen slightly obliquely in this view.

The DV projection (Figures 1A, 2A) of the normal lung shows symmetry in the volume with the impression of the lungs not quite reaching the periphery of the shell, an artifact of a 3D structure displayed as a 2D image combined with the lungs lying dorsal to the widest diameter of the shell. The lung volume is symmetrical. The main bronchus is seen coursing from cranial to caudal in the medial lung field at the approximate level of the ribs as they insert on the pleural bones and is curvilinear on this view, coursing caudomedially. The horizontal soft tissue opaque striations of the branching channels are observed coursing from the mainstem bronchus to the periphery perpendicular to the main bronchus. The opacity of the lung is symmetrical and uniform on both sides and the margins of the lung are smooth occasionally with mild undulations caudally, and the peripheral pleura is uniform in thickness. The lung is always superimposed on the other coelomic organs on this projection.

The RoCd projection (Figures 1B, 2B) for normal lungs will show the lungs' cross-sectional area as ~40 ±10% of the shell internal volume with symmetry in volume between sides. The lung margins should be smooth ventrally and conforming to the shell laterally and dorsally with the lungs meeting on midline medially. The main lung bronchus accompanied by a vein (ventral) and an artery (dorsal) is located ventromedially in each lung with clear conspicuity of margins of these structures. Extending out from the main bronchus, the lung parenchyma has a honeycomb or reticular appearance due to the channels that branch from the mainstem bronchi and their associated niches being essentially end-on on this projection. The pattern and opacity of the lung lobes are uniform with symmetry between sides. This view is particularly helpful for assessing the symmetry of volume for each lung lobe and overall lung volume.

The left (Figures 1C, 2C) and right (Figures 1D, 2D) lateral projection for normal lungs will show each lung lobe occupying ~40 ± 10% of the shell internal cross-sectional area. The lungs each taper toward the caudal and have uniform opacity. The main bronchus is seen coursing from cranial to caudal, horizontally through the ventral lung field. There are vertical striations through the lungs on these views that reflect the channels that branch from the main bronchus. The margins of the lungs are smooth. These views are particularly helpful for determining if gastrointestinal distension or carapace pathology is present as a cause for altered lung volume.

**Figure 3:**
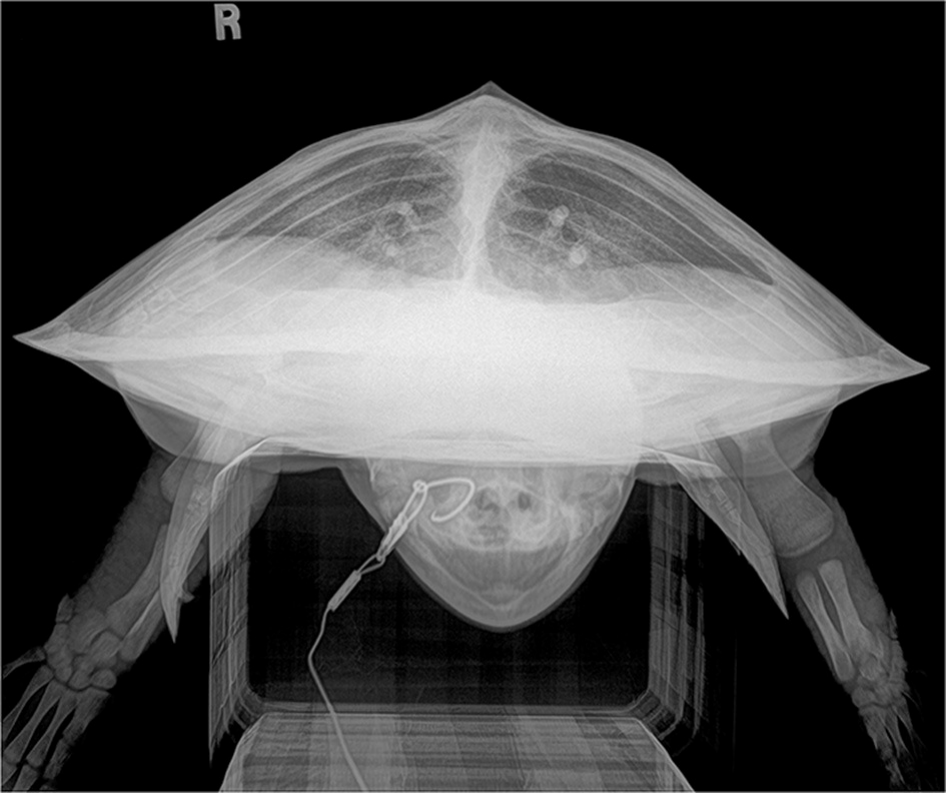
Rostrocaudal (RoCd) projection of a hook-and-line capture, otherwise healthy juvenile Kemp's ridley sea turtle (*Lepidochelys kempii*). The lung fields are asymmetric with the right lung field hypoinflated and reduced in volume slightly when compared to the left lung field due to non-uniform gas accumulation within the GI tract. The lung fields fill ~40% of the coelomic cavity. Both the left and right main bronchus, pulmonary arteries (dorsal), and pulmonary veins (ventral) are evident. This radiograph represents a normal variation.

Normal variations seen included mild asymmetry of lung fields on DV, RoCd, and lateral projections that could be attributed to asymmetrical limb position and/or gastrointestinal content, causing the compression of lung fields as seen in Figure 3.

## Discussion

Radiography is an economical and accessible tool for assessing the lower respiratory tract in sea turtles in rehabilitation. Given that respiratory disease is a prevalent problem, and clinical signs are not always apparent or specific, radiography is a critical tool for rehabilitation and recovery centers to obtain essential information about the health of their patients. Therefore, understanding normal pulmonary radiographic findings is essential to diagnose abnormal pathology.

While the caudal coelomic organs are frequently challenging to assess radiographically due to poor inherent detail, the high inherent contrast of normal lungs allows adequate evaluation in most cases. Utilization of the four projections described is needed to adequately assess all lung fields with the RoCd and DV projections particularly helpful for evaluating lung symmetry. While the DV projection helps assess lung symmetry, it does not allow for an in-depth evaluation of the lung parenchyma due to the superimposition of all the other organs. The accuracy of assessment is affected by rotation, and careful positioning is necessary to ensure images are optimized. Very subtle lesions may be missed on radiography, but CT scanning is not financially viable in every case in the rehabilitation setting. Both financially and clinically, it is preferable to release animals as soon as they are ready, and acquiring baseline radiographs at the time of intake can help determine quickly if animals have comorbidities that require therapy, or can be treated, monitored, and released alongside the clinical examination and other diagnostics. Cold-stunned sea turtles, in particular, are known to be at high risk of pneumonia, osteomyelitis, and other infections that may be present before entering or during rehabilitation (2). Quickly identifying those turtles with pulmonary lesions *via* radiography can help speed recovery and potentially limit the spread/risk to others by keeping healthy and non-healthy groups separated.

While other studies have shown the relative benefits of using CT over radiography in turtles, this is not financially viable in most rehabilitation settings for every turtle (4). The advent of digital radiography has greatly improved the image quality and sensitivity of radiographs for turtle pathology over traditional analog radiography. The superimposition of structures and the presence of the shell will always limit radiography, but optimized radiography remains a vital tool in turtle rehabilitation for screening and diagnosis of respiratory disease.

This study details the normal radiographic observations of the lower respiratory tract of Kemp's ridley sea turtles *via* digital radiography and will be a helpful reference for evaluating radiographs of juvenile Kemp's ridley sea turtles in rehabilitation.

## Data availability statement

The original contributions presented in the study are included in the article/supplementary material, further inquiries can be directed to the corresponding author.

## Author contributions

CB: organization, writing, and editing. DM: introduction, review, and editing. AL and SD: results, review, and editing. All authors contributed to the article and approved the submitted version.
